# Care for Language: Etymology as a Continental Argument in Bioethics

**DOI:** 10.1007/s11673-021-10125-z

**Published:** 2021-10-01

**Authors:** Hub Zwart

**Affiliations:** Dean Erasmus School of Philosophy, Erasmus University Rotterdam, Bayle Building, Room J5-65, Burgemeester Oudlaan 50, 3062 PA Rotterdam, The Netherlands

**Keywords:** Continental bioethics, Etymology and ethics, Philosophical methodology, Continental philosophy of language

## Abstract

Emphasizing the importance of language is a key characteristic of philosophical reflection in general and of bioethics in particular. Rather than trying to eliminate the historicity and ambiguity of language, a continental approach to bioethics will make conscious use of it, for instance by closely studying the history of the key terms we employ in bioethical debates. Continental bioethics entails a focus on the historical vicissitudes of the key signifiers of the bioethical vocabulary, urging us to study the history of terms such as “bioethics,” “autonomy,” “privacy,” and “consensus.” Instead of trying to define such terms as clearly and unequivocally as possible, a continental approach rather requires us to take a step backwards, tracing the historical backdrop of the words currently in vogue. By comparing the original meanings of terms with their current meanings, and by considering important moments of transition in their history, obfuscated dimensions of meaning can be retrieved. Thus, notwithstanding a number of methodological challenges involved in etymological exercises, they may foster moral articulacy and enhance our ability to come to terms with moral dilemmas we are facing.

## Introduction: the Linguistic Turn in Continental Bioethics

Emphasizing the importance of language is a key characteristic of philosophical reflection in general and of bioethics in particular. Analytic philosophy is known for its emphasis on clear definitions and rigorous arguments, up to the point of trying to create a logically perfect language, neutral vis-a-vis metaphysical world-views and freed from the ambiguities and historical heritages pervading everyday grammars and vocabularies (Beaney 2013). Continental philosophy may likewise boast a long tradition of concern for language, although here the aim is not to eliminate the historicity and ambiguity of language but rather to make conscious use of it (Lafont 2015; Culbertson 2019), for instance by closely studying the history of the key terms we employ in bioethical debates. As Catherine Mills (2010) argued in an editorial in this journal, during past decades, bioethics remained fairly “resistant” to critical incursions from continental approaches, such as phenomenology or poststructuralism, opting for “protectionism” instead. This paper outlines what etymology as a continental contribution may add to contemporary bioethical debate.

A continental approach to bioethics will pay due attention to the history (the diachronic dimension) of moral language, I will argue. Continental bioethics entails a focus on the historical vicissitudes of the key signifiers of the bioethical vocabulary, urging us to study the history of terms such as “bioethics,” “autonomy,” “privacy,” and “consensus.” Instead of trying to define such terms as clearly and unequivocally as possible, a continental approach rather requires us to take a step backwards, tracing the historical backdrop of the words we currently use. In order to grasp the meaning of the term “ethics,” for instance, it makes sense to start with the original meaning of words such as ἦθος and ἠθικός in ancient Greek. Notice that the word “term” itself comes from the Latin noun *terminus*, indicating that a “term” is the temporary end-point of a long linguistic journey.

Studying the history of key signifiers entails an etymological disambiguation or even “reduction,” albeit in the literal (etymological) sense, for “reduction” literally means “bringing back” (“reducere” in Latin) multiple pathways of meaning. The historical development of key bioethical signifiers is not necessarily seen as progress (resulting in increased clarity for instance). Rather, etymological exercises will often reveal instances of loss, forgetfulness, and erosion of meaning. What is obfuscated in contemporary usage may actually prove quite valuable and point to lost aspects of meaning which may help us to come to terms with contemporary dilemmas. Thus, the etymological turn in bioethics often entails an exercise in retrieval. The ultimate aim of an etymological detour is not to contribute to language studies but to enlighten current moral challenges, allowing us to address normative dilemmas emerging today more adequately, by enhancing our sensitivity to linguistic nuances and complexities. Thus, the etymological argument substantiates the claim, put forward by Mills (2010), that continental philosophy may revitalize bioethics discourse, bringing it closer to lived experience.

We may start our endeavour by applying the etymological argument to (the history of) the (fairly recent) term “bioethics” itself. Introduced in 1927, the term initially referred to responsibilities of humans towards animals, plants, and the biosphere (Sass 2007; Martensen 2001). When the term was reinvented, more than four decades later, at Georgetown University in 1970, however, the focus shifted towards biomedical challenges. And this ambiguity (this wavering of bioethics between life sciences ethics on the one hand and biomedical ethics on the other) is still noticeable today. This should not necessarily be considered a weakness, however. Rather, it may point to the awareness that moral principles and dilemmas emerging in biomedical ethics should not be disconnected from considerations concerning the responsibilities of humans vis-à-vis the biosphere (as happens when medical ethics and environmental ethics become compartmentalized).

## Etymology in Continental Philosophy

There is a long tradition of etymology as an argument in moral philosophy, notably involving the oeuvres of continental philosophers. This applies, for instance, to Friedrich Nietzsche, who was trained in philology before turning to philosophy. In *Genealogy of Morals*, for instance, Nietzsche (1887/1980, first essay, §4) famously points out that, originally, the terms “good” and “bad” (“schlecht” in German) had a different meaning than in modern times, standing for “exceptional” (noble) and “mediocre” (vulgar, ordinary) respectively. Thus, someone who obediently follows the rules will be considered “good” today, Nietzsche argues, but would have been considered “bad” (“schlecht,” inconspicuous) in the past. This shift of meaning is not completely lost to us. When reading a biography of, say, Lou Reed (Bockris 2014), we may see the protagonist as remarkable and outstanding, even if we at times consider his behaviours towards others as morally problematic. Apparently, both standards of moral quality are still available to us, even if they seem mutually incompatible.

Martin Heidegger is likewise a prominent example of a continental philosopher who made ample use of etymology in his work. In *Being and Time*, he famously questioned the traditional interpretation of “truth” as correspondence (*adequatio* in Latin) between *thinking* and *being*, or, in more modern terms, between theoretical conjectures and empirical observations. Heidegger points out that the Greek term for truth (ἀλήθεια) had a different meaning. The privative *α*- suggests that something had been forgotten which is now being revealed, retrieved, or disclosed, resulting in Heidegger’s rival understanding of truth as “un-forgetfulness.”

Along similar lines, in *What is called thinking?* Heidegger (1954/2002) points out that, although terms like “method” and “methodology” are often identified with rigid research protocols, notably of the experimental type, the literal (etymological) meaning of “method” rather suggests that it is something that is developed *along the way* (μετ᾽ + ὁδός in Greek, where ὁδός means “road”). From an etymological point of view, method means openness, a willingness to reconsider the path we have explored in retrospect (Zwart 2020). To the extent that etymology is a “method” for bioethics, it is a method precisely of this kind: tracing the intricate path of concepts through history, in retrospect. Etymology means “vade retro,” taking us back to the beginning, to find out what was gained and lost along the way.

This sensitivity to language and its history is a core feature of continental thinking, from Hegelian dialectics (e.g., Hegel’s conscious use of the ambiguities of key dialectical signifiers such as “Aufhebung”) via Nietzsche and Heidegger up to Freudian and Lacanian psychoanalysis. Freud (1910/1943) for instance, building on the work of linguist Karl Abel, stressed that primordial words (“Urworte”) remarkably often had antithetical meanings, a phenomenon which is still noticeable today. Freud gives some (notably German) examples of this, such as “stumm” (voiceless) and “Stimme” (voice) but also *bat* (“good” in Old Saxon) and *bad* (which has the opposite meaning in contemporary English). According to Freud, in phenomena such as dreams, this basic ambiguity of primordial words is still retained and exploited. This line of research was taken up and elaborated further by Jacques Lacan, who combined Freudian psychoanalysis with structural linguistics (De Saussure 1916/1968) in paying much attention to shifting relationships (“displacements,” including reversals) between *signifiers* (i.e., typographical words or sound patterns) and the concepts *signified* by them (Gillett 2015; Zwart 2016).

Building on Heidegger, chronic discontent in established discourse, seeing it as profoundly questionable, was taken up by later authors, for instance Jacques Derrida, who introduced key terms such as “deconstruction” and “différance” in order to move way from a language he considered tainted by a logic of binary oppositions. The term “deconstruction” plays on Heidegger’s concept of “destruction,” i.e., the effort to reveal the original meaning of terms by eliminating the sediments of established ontological interpretations (deconstruction = de-*con*-struction), while différance is a deliberate misspelling of *difference* (although the two words are pronounced identically), indicating that words can never be defined exhaustively, but only by appealing to other words, from which they *differ*, so that their meaning is forever *deferred* through endless chains of signifiers. A similar attitude to language can already be encountered in Jacques Lacan, who translated Heidegger’s essay *Logos* (Heidegger 1951/2000) and coined the term *nom-du-père* (pronounced in the same way as *non-du-père*) to emphasize how the prohibitive function of (God) the father inaugurates the symbolic order. Another example of Lacan’s use of language is *extimacy* (something which is both external or foreign and intimate) in the context of organ transplantation and intimate technologies (Zwart 2017, 2019). Rather than opting for an etymological detour, however, these authors destabilize and question established meanings by adding or replacing components to key signifiers (e.g., *différance* instead of difference or *extimate* as a combination of external and intimate), thereby questioning the binary logic of traditional ontologies in a playful manner. Thus, although the motives are similar (distancing ourselves from the pitfalls of established discourse), the technique employed by the etymological argument is a slightly different one (tracing the history of terms back to the moment of commencement). Yet, the etymological argument is not absent in their work. In a treatise on religion for instance, Derrida (1996) indicates how the etymology of the term “religion” reveals two antithetical meanings, namely *to bind to* (to hold back) versus *to relink* (with something different, e.g., in the case of conversion), an etymology which reveals two apparently contradicting roles (conservation versus disruption) which religion may have.^1^

I will now elucidate the relevance of etymology for bioethics, focusing on a number of key signifiers (although in principle the etymological approach is applicable to *any* bioethical concept), namely: *naturalness*, *autonomy*, *consensus*, and *privacy*.

## First Example: Observing Naturalness

An interesting example of the relevance of etymology in bioethics is the verb “to observe.” At first glance, this seems to refer to empirical activities, e.g., “observing nature.” On closer inspection, however, it is clear that this verb may also have a more normative meaning, even in modern languages, e.g., “to observe a rule.” The connection between these two meanings seems lost to us, but etymology provides the bridge. Initially, the Latin verb *observare* meant to *heed*, to *serve*, and to *respect* nature. An observer of nature was a *servant* of nature, was *serving* nature, studying nature carefully for normative hints and guidance. This is also suggested by the famous Hippocratic view of a physician as a *minister naturae* (Demaitre 2013, 15; Bergdolt 1999/2008, 91), a servant (assistant) of nature, whose interventions must be attuned to nature (must “observe” nature), to allow nature to do her wholesome work. Observing nature was not a neutral activity but rather an activity which instilled awe and respect for nature. The objective of “*observare*,” in the original sense of the term, was to discern order and harmony in nature, so that humans could live and act in adherence to this order. Thus, nature was seen as providing norms we should follow, building on the basic conviction that what is “natural” is “good.”

In the course of modern history, this dimension was eliminated, and the time-old association between “natural” and “good” became disconnected, so that “to observe” evolved into a detached, empirical activity: observing nature as “objectivity.” Indeed, modern ethics (Kant, Bentham, etc.) aimed to segregate the natural from the normative. As nature became disenchanted, the empirical and normative dimensions of the verb “to observe” became disconnected. Observing natural processes seemed quite different from observing rules. In bioethics, this segregation was endorsed by many, and naturalness has been vehemently rejected as a valid argument in moral deliberations. Trying to derive norms from nature allegedly equalled falling into a trap known as the naturalistic fallacy (Zwart 1994).

In everyday deliberations, however, the idea that what is natural is good can still be encountered, up to this day. Outside bioethics, in everyday discourse, the label “unnatural” is often used to indicate that something (e.g., GM food) is morally questionable. This evidently results in a tension between expert bioethical discourse (where naturalness as an argument tends to be discarded) and public debates (where “naturalness” continues to function as an argument in debates about food, health, and the environment).

An example of this, taken from the debate about genetic modification of animals and crops, is the argument that traditional forms of hybridization (employed by humans since the Neolithic revolution) are allegedly more “natural” (less “unnatural”) compared to genetic modification techniques (e.g., knocking out or adding genes). Yet, etymology informs us that the word “hybrid” already entails an element of caution or even normative disapproval. It is derived from the Greek term hubris (ὕβρις) which means excessive self-confidence: an insolence against the divine order. Apparently, there is already a signal of warning against hybridization embedded in language itself (Meilaender 2020). In other words, notwithstanding the struggle of the Enlightenment (represented by philosophers such as Kant and Bentham) to disconnect the natural from the normative, this struggle still continues, subliminally at least. And in certain moral traditions, e.g., Catholic (Thomist) ethics, the argument that natural equals good can still be encountered. These examples indicate that something in the claim that what is “natural” is “good” still deserves to be taken seriously. Discarding it on “rational” grounds will not only impoverish our moral vocabulary but will also result in discontent and a return of the repressed (via displacements, so that other terms, e.g., “sustainability” or “health” are used instead of naturalness, to circumvent the taboo).

## Second Example: Autonomy

Autonomy is seen by many as a key principle and core value in bioethics, notably in medical ethics (Childress 1990; Varelius 2006). What is less well known is that this term has a long and convoluted history that goes back to ancient Greece. Sophocles’ *Antigone* is the text where the signifier “autonomy” (αὑτ ό νομος) is used for the very first time (Zwart 1993, 2018, 783), a fact which confirms the relevance of consulting ancient tragedies in the context of contemporary debates (Gillet and Bowyer 2014; Gillett and Hankey 2014). Antigone, the tragedy’s heroine, is not making her own free choice in the modern (liberal) sense of “self-determination,” however. Rather, while confronting top-down political morality (the “human” law), she claims the right to act in accordance with an unwritten, divine law. Her fatal (and extremely unprofitable) craving to adhere to this “unwritten” law is imposed on her by what can be considered as a summons, a calling, a vocation, speaking from within, but coming from “elsewhere”: a silent voice, which nonetheless cries out, a decisive instigation, resulting in her fatal *passage à l’acte*.

For contemporary readers, it has become difficult to fathom the precise nature of Antigone’s divine calling because the signifier *autonomy* has been affected by a series of rather drastic displacements. If we follow its trajectory diachronically (through history) it is clear that nowadays the term no longer has the same meaning (no longer refers to the same “signified,” linguistically speaking) as it did in ancient Greece (Zwart 1993). For Kant, for instance, “autonomy” means acting in accordance with the law of reason, which is already quite unlike the opaque, unfathomable (“irrational”) divine injunction (demonic and heathenish if you like) to which Antigone commits herself. And contemporary morality takes this even one step further. In contemporary debates, “autonomy” often acquires a rather liberal meaning, indicating the right to act in accordance with one’s own predilections, however “eccentric” these may be, to use a term that was used by John Stuart Mill (1859/1974). In other words, in contemporary bioethics, the ancient association, the etymological legacy which connects the signifier “autonomy” with divine calling, has become eclipsed or obfuscated. As Maartje Schermer (2002) explains, in current bioethical discourse, “autonomy” is now generally understood as (and equated with) the patient’s right to self-determination within the context of medical and research practices. It has become a *negative* right: the right to non-interference, to making decisions concerning your own life without being controlled by others, thus protecting patients from unwelcome interference by physicians and other healthcare professionals. In this short-circuiting between autonomy and self-determination, however, something has been lost, namely the *positive* (literal) dimension, for autonomy also means responsiveness to a norm, the conscious endorsement of a norm, a world-view even.

Yet, this forgetfulness is far from absolute. In contemporary moral collisions, in bioethical tragedies, certain unsettling aspects may resurge which suggest that there is more to autonomy than mere self-determination and that even in the secular present, human individuals may be driven by opaque imperatives which may prove harmful or even detrimental (rather than benign and beneficial) to themselves. Antigone may be considered an “autonomous” subject but not in the sense of a modern *calculating* subject. The voice from “elsewhere” which addresses her may not be so readily articulatable in terms of standard bioethics discourse. It may be something quite difficult to accommodate from a standard bioethical view. The etymological detour increases our sensitivity to the fact that there may be more to autonomy than mere self-determination.^2^

This explains what in linguistics (and in psychoanalysis) is referred to as “displacement,” i.e., the tendency to replace loaded terms (such as “autonomy”) with substitutes which seem less burdened by cultural heritage, such as “self-determination”: apparently a smoother, less commanding alternative for “autonomy.” The latter is a “heavier” term, “tainted” if you like by history. According to Nunner-Winkler (2008), for instance, while self-determination refers to an independent, informed opinion about important aspects of our life, autonomy is a more demanding term insofar as orientation-providing norms and a conscious justification of their validity come into play. From the perspective of continental bioethics, however, such layers of meaning must be kept in mind. In cases of tragedy, these other (older) layers of meaning may suddenly resurge, so that etymological awareness may prove highly relevant in addressing contemporary moral conflicts, notably of the tragical kind. Rather than solving the dilemma, etymology may make us more sensitive to the complexities involved.

## Another Example: Consensus

Another example is the term “consensus” which, at first glance, may seem a purely neutral term. Indeed, according to authors such as Tristram Engelhardt (1986), “consensus” is part of a bioethical “lingua franca,” our “neutral” common language. From an etymological perspective, however, even the term *consensus* is not as neutral as it may seem. Historically, a connection can be discerned between the (allegedly neutral) signifier “consensus” and a particular world-view, a particular moral tradition, in this case Protestantism (Zwart 2001). The signifier “consensus” is loaded with historical reminiscences. The *Consensus Tigurinus* (Zurich consensus, 1549) and the *Consensus Pastorum Genevensium* (Geneva consensus, 1551), for instance, refer to crucial meetings organized during the early days of the Reformation in order to achieve consensus among theologians in challenging and potentially disruptive disputes. These consensus conferences were deemed necessary because a central magisterium was no longer available. For protestant theologians, the papal Holy See could no longer play that role. This historical legacy (this practice of linking the term consensus with a certain locality) seems to reverberate in bioethics, for instance in the case of the *Appleton consensus* (1988), involving a group of physicians and bioethicists from ten different countries who met at Appleton, Wisconsin, to discuss guidelines for decisions to forego medical treatment (Stanley 1989). Many additional examples of consensus statements can of course be given, from the *Consensus statement of the Society of Critical Care Medicine’s Ethics Committee* regarding futile and other possibly inadvisable treatment (Society of Critical Care Medicine 1997) via the *Consensus statement on medical ethics and law for doctors of tomorrow* (Vivekananda-Schmidt and Hooper 2020) up to the AHA/ACC *Consensus statement on medical professionalism and ethics* (2020). The basic structure tends to be similar and recognizable, namely an effort to reach agreement on sensitive moral issues among professionals in the absence of top-down, pre-established norms.

The genealogy of the term does not disqualify its current use or relevance as such, of course. What is questionable is not the validity of the concept (as an element of a particular conceptual grid) but its alleged neutrality. The signifier *consensus* conveys a particular way of framing the issue, a particular (and therefore questionable) manner of posing questions, of structuring problems (“questionable” in the non-pejorative sense of the term). The etymological argument allows us (and urges us) to become sensitive to this. One of the performative implications of adopting the term “consensus” is that we sacrifice the quest for truth (e.g., the truth of Catholicism) to opt for “mere” consensus instead. Consensus means consensus among experts, attained in accordance with a particular methodology (e.g., moral deliberation, reflective equilibrium) and is by definition of a temporary nature.

## Final Example: Privacy

Let me add another example, namely the signifier “privacy,” to elucidate the relevance of the etymological approach. In mainstream bioethics, *privacy* is regarded as something positive, a basic human right, something good which deserves to be protected. The etymology of the term, however, should raise some suspicion. Etymologically speaking, privacy comes from “privation” (“bereavement,” *privatio* in Latin), a pejorative term, a *negative* signifier, indicating a lack. The term *privation* suggests that the individual in question misses something, is deprived of something, e.g., care and attention by others. It suggests living in seclusion, outside the circuits of solidarity and sharing. Somehow, a transvaluation of values has occurred, changing a negative term into a positive one because nowadays, by replacing *privation* by *privacy*, this negative term, indicating absence (*privation*), has been reverted into something positive (*privacy*), a right or good which ought to be protected. In short, a displacement has occurred from *privation* (negative, “bad”) to *privacy* (positive, “good”).

Privacy sets limits to social meddling. It is not a neutral term, however, but conveys and promotes a particular *image* of human beings, seeing them as disconnected autonomous atoms, rather than as interconnected elements in symbiotic sociocultural ecosystems. This is precisely where privacy differs from confidentiality, I would argue. For although both terms at first glance may seem quite similar, confidentiality literally means to entrust sensitive information to someone (e.g., a physician) whom we trust (etymologically speaking, confidentially builds on *fidere* “to trust”). Thus, while confidentiality presupposes a personal trust relationship, privacy is a more abstract and anonymous term, connected with responsible data management, for instance in the context of genetic testing, where regulating the relationship between insurance companies and their clients is based on distance or even distrust rather than trust (Pugh 2021). Although etymology does not entail clear arguments for or against the importance of privacy, it fosters perceptivity for aspects which seem lost in its standard use, as one of the key signifiers of mainstream bioethics. Building on these examples, I will now outline how the “etymological argument” works out in practice.

## The Etymological “Argument”

The etymological argument consists of a number of steps:

- (a) The starting point is the intuition that a particular term is used improperly or inauthentically in current discourse, in the sense that it has drifted away from its original meaning, even up to the point of acquiring connotations that seem juxtaposed to the initial ones. Important aspects of meaning seem to be neglected. In other words, the etymological detour is often fuelled by a sense of discontent in contemporary deliberation: the awareness that we somehow lack the moral articulacy to capture the true complexity of a moral problem situation. Something of importance may have been lost or may have been neglected.
- (b) Subsequently, the etymological argument builds on the conviction that the proper (genuine) meaning of the term can be retrieved by tracing its history, notably by recollecting it original meaning.
- (c) This original meaning has been dissimulated and obfuscated (obscured, forgotten) in current usage. In order to retrieve the original meaning, we must bracket the current (established, inauthentic) meaning and investigate the origin and history of the term.
- (d) Finally, by comparing the original term with its current meaning, and by considering important moments of transition in the history of the terms we use, these obfuscated dimensions can be recollected.

Thus, by studying the history of terms (seen as temporary end-points), eroded aspects of meaning (discarded or obfuscated during previous stages of deliberation) may be retrieved. While an analytic philosophical approach aims to reduce meaning *per via di levare*, i.e., by taking away (by consciously removing ambiguous layers of meaning), a continental approach rather uses etymology to expose and explore the basic ambiguities at work in the terms we employ, often without being conscious of them. In principle, awareness of multi-faceted aspects of meaning is considered a strength, for it will make us better listeners and readers. In short, it will enhance our basic ethical skills.

This does not imply that we should by definition endorse or privilege the past over the present. Quite the contrary, etymological inquiries may also have the opposite result, namely of showing exactly where and when problematic connections emerged which are still challenging us. To give a concrete example, the word “organ” means *tool* or *instrument* in ancient Greek. The identification of bodily organs with “tools” seems supportive of understanding the human body as an aggregate of replaceable parts: of partial objects (organs) rather than as an organic whole (Zwart 2019). This deceptive ontological association may give rise to an underestimation of the detrimental impact of transplantation medicine on the integrity or wholeness of the body (Sveneaus 2010; Heidegger 1992). Notice that *integrity* literally means “wholeness” in Latin. In this case, etymology may help to problematize the instrumentalization of organs. The result of an etymological detour in this case is not that transplantation medicine as such should be rejected. Rather, it fosters sensitivity to some of its side-effects, to the collateral damage of organ transplantation as a disruptive intervention, in the aftermath of which bodily integrity will only partially be restored (Leder 1999; Nancy 2000; Sveneaus 2010; Zwart 2019).

In short, etymological exercises may prove a complicated challenge. First of all, etymology often guides us back to ancient Greek and Latin, as we have seen. And although many contemporary scholars may still possess some basic knowledge of these languages, the vast majority will no longer be fluid in them and therefore no longer sensitive to all the nuances of meaning once at work here. Heidegger himself already emphasized this when he desperately tried to retrieve the meaning of pre-Socratic sayings. We no longer *hear* the sound of words like ἀλήθεια. We no longer literally hear how such words must have sounded when they were spoken long ago, for instance in Socratic dialogues or in the dialogue between Christ and Pilate (John 18:38). Even if we remember the term, relying on “dead” written documents, we will probably miss something (e.g., the living phrase, the particular moral dialect that was used). This does not imply that etymology is an impossible objective. Rather we must see etymological “reduction” as an *interminable* analysis, to use the Freudian phrase.

If etymology is such a difficult or even “questionable” exercise, in the non-pejorative sense of the term—namely in the sense that, eventually, completion may never be achieved—the question may rightfully be asked why we should dive into this at all? Isn’t there an easier or more reliable alternative to care for language? Shouldn’t we opt for unequivocal definitions, for instance, as analytic philosophers would argue, allowing us to rid ourselves of all historical sediments of meaning? Various strategies can be applied to forgo linguistic ambiguities, such as formalization, replacing everyday language (with its opaque intuitions, etc.) by rigorous logic, complemented perhaps by quantification, by an empirical (social science) approach (providing us with verifiable facts). Indeed, combining clear definitions with hard facts, wouldn’t that be more helpful to solve our problems?

The etymological argument entails, however, that eventually, such alternatives will not really help us. Genuine dilemmas and tragic collisions will continue to fuel discontent in linguistic straitjackets, bent on eliminating complexity. The removal of ambiguity will eventually give rise to a return of the repressed. The straitjacket of rigorous definitions will result in discontent in apparently consistent argumentations. When faced with tragic dilemmas, we are bound to experience the poverty of our language, our lack of moral fluency and literacy, hindering us to articulate important experiences that seem to escape us, so that logical analysis, consistent as it may be, may be experienced as a violation of the complexity of the situation we are facing. This is a debate which has haunted bioethical discourse from the very start (from the 1970s onwards), and my aim is not to solve it in this paper. What I do want to point out is that the analytic approach (relying on clear definitions and rigorous argumentation) is not the only alternative available. There is another, more “continental” approach to this, where ambiguity and complexity are celebrated rather than eliminated.

## Conservatism

Another possible objection against the use of etymology in bioethics is that it seems inherently conservative. The act of guiding us backwards into history (*reduction* in the literal, etymological sense of the term) questions the modernistic idea that history equals progress. Tracing the history of terms from primordial commencement to present meaning does not necessarily foster a conservative attitude, however. Rather than entailing a nostalgic craving to return to the past, the etymological argument summons us to reconsider the meaning of the terms we use *under present circumstances*. Rather than seeing history in terms of linear progress or decline, the vicissitudes of moral signifiers will often reflect a fairly dramatic curve. Initially, a word may be introduced without consciously and explicitly exploring and addressing all the ambiguities involved. At a certain point, internal tensions may increase, giving rise to confusion or even conflict, and this may force us to redefine the term at hand, resulting in a process of catharsis (linguistic cleansing). The etymological argument entails, however, that the terms we use will continue to carry the scars of these previous events, these previous conflicts and cleansings. Their history is somehow retained in them. This explains why the etymological argument is notably meaningful when facing complex dilemmas. In a critical situation, the inherent conflicts embedded in our language tend to be re-invoked.

Here, it may again be helpful to give a concrete example, this time from outside the bioethical realm. In the United States, the 2020 presidential election raised the question: *is this* democracy? From a continental philosophical perspective, such a question urges us to take a step backwards: *what is* democracy? And the etymological argument encourages us to *go back* and to *return to* the original meaning of the term “democracy,” so as to become sensitive to what may have been lost. Etymology does not provide solutions. Yet, if skilfully used, it may add to the depth and quality of the analysis and even increase the quality of the solution that is eventually reached. By exploring the history of terms, we may retrieve and revivify their meaning under present circumstances. For instance, by asking the question how “democracy” relates to “populism,” two terms which, etymologically speaking, have similar meanings more or less, being derived from δῆμος (Greek) and *populus* (Latin) respectively (Klink, Jansen, and van der Geest 2020). Why, then, are they so often juxtaposed? Rather than fuelling a longing for democracy as it was understood and practiced in ancient Athens, the etymological argument summons us to become more attentive to exactly *how* we use the word “democracy” *today*.

## Conclusion

It has been argued that continental approaches are under-represented in contemporary bioethics. This paper presents the claim that a continental approach to bioethics notably entails fostering our sensitivity to the historical dimensions of moral language. I have argued that an etymological detour reveals (retrieves) the inherent complexities and ambiguities of our key bioethical signifiers. Etymology may be considered a bioethical “method,” albeit not in the sense of a strict protocol but rather as a path to follow. Etymology may be considered as a bioethical “argument,” albeit not in the propositional sense of the term but in the sense of *making clear* (“arguere”) that the words we use are more open to the inherent complexities of the dilemmas we are facing than their straightforward definitions seem to suggest. Etymology will reveal rather than reduce complexity. After an etymological detour, we may decide to adopt one possible meaning rather than others but now in the form of a conscious decision. Etymological detours inform us that established definitions of bioethical signifiers benefit *one particular* interpretation at the expense of others. These decisions and interpretations are inherently questionable, however, so that etymology enables us to question them, by proposing alternative options. Etymology reveals the intimate relationships between words and actions. We use established definition to legitimize established courses of action, and etymology may come to our aid in situation of reluctance, discontent, or doubt, by fostering moral literacy and diversity and strengthening our fluency in multiple moral dialects.

## Footnotes

1 As to Lacan, in a comment on Augustine’s *Confessions*, he points to the etymology of the word *infant* (= unable to speak) for instance, to elucidate the moment when a child enters the symbolic order of language.

2 This was also captured by Martin Luther, for instance, when he stated, “Here I stand, I can do no other” (not coincidentally followed by the phrase “God help me, Amen”).
